# Two Cases of Medico-Legal Interest

**Published:** 1889-02

**Authors:** H. N. Moyer

**Affiliations:** Chicago, Illinois


### TWO CASES OF MEDICO-LEGAL INTEREST.

BY H. N. MOYER, M. D., CHICAGO, ILLINOIS*

During the past summer I was requested by the coroner to make a post-mortem examination of the remains of a person upon South Jefferson street, in this city. The body was that of a man about fifty years of age, fairly well developed, and remarkably free from decomposition, considering the then prevailing high temperature and the fact that death had taken place several days before.

On inquiry among the friends of the deceased, I learned that the body had been sent from Watertown, Wisconsin, and the physician in attendance, at the time of death, had certified that poisoning was the immediate cause. On removing the clothing

*Read before the Chicago Pathological S c'ety, December 10, 1888.

Poisoning with paris green ✓

a perforating gun-shot wound of the chest was found at the left border of the sternum in the third intercostal space. The bullet had penetrated the inner border of the left lung and lodged in the wall of the pericardium. Here lymph had been thrown around the foreign body and it was partially encysted. A deep ecchymosis was found upon the left border of the heart at the junction of the auricle with the ventricle. Apparently very little disturbance had been caused by the injury. No inflammation, hemorrhage, or pus, and the process of repair was taking place rapidly. The abdomen was retracted and not the slightest sign of decomposition was observable. The intestines were of a dark mahogany color, presenting a strong contrast to the yellow fat of the omentum. The stomach presented little change on its intestinal surface, the mucosa deeply injected, softened and velvety. Its surface was covered with a green substance, which, upon analysis, proved to be paris green. A two-ounce vial was nearly filled with the salt after washing the stomach and intestines.

The quantity of the poison precluded the idea of homicide, and eventually gave us the true solution of the mystery. It seems there had been a neighborhood row, and the deceased had fired from a window at his son-in-law, the latter, in haste to get away, had fallen backward just at the moment the pistol was discharged. The deceased then turned the pistol toward his own breast and inflicted the wound described. This attempt at suicide proving futile he fled, and some days later, doubtless believing himself a murderer, he took the poison with fatal effect. This is one of the largest doses of arsenic ever taken, amounting in all to nearly five ounces.

*Case II.*—A man was found lying in a depression, some three feet in depth, between the curbstone and sidewalk, in an unconscious condition. The bottom of this depression was formed by loose dirt. Death took place some four hours afterward without return of consciousness. The body was that of a middle-aged man, well developed. *Rigor mortis* was well marked, and there were no external marks of injury or violence. On reflecting the scalp a not well defined ecchymosis (in shape, about one and a half inches in diameter, and irregular) was found on the vertex.

A careful examination of the brain, and other internal organs was made, bt they all appeared to be perfectly normal. As the structures of the neck were removed a small ecchymosis was found in the posterior wall of the pharynx, which led to a careful examination of the parts in this region. A slight inequality in the body of the fifth cervical vertebra was noticed, and when the head was grasped and tilted backward, a complete transverse fracture of the body of the fifth cervical vertebra was brought into view.

The circumstances in this case pointed to the possibility of homicide, but the opinion was expressed that such an injury could hardly have been produced by homicidal violence, in the absence of more distinct external marks of injury. Further, that the fracture was probably caused by a fall from the sidewalk down the slight depression, where the patient was found. Upon this opinion proceedings were discontinued.

